# Association between telomere length and neuropsychological function at 4–5 years in children from the INMA project: a cross-sectional study

**DOI:** 10.1007/s00787-023-02361-y

**Published:** 2024-01-22

**Authors:** Irene Campos-Sánchez, Eva María Navarrete-Muñoz, Miriam Hurtado-Pomares, Jordi Júlvez, Nerea Lertxundi, Dries S. Martens, Ana Fernández-Somoano, Isolina Riaño-Galán, Mònica Guxens, Jesús María Ibarluzea, Tim Nawrot, Desirée Valera-Gran

**Affiliations:** 1 Department of Surgery and Pathology, Miguel Hernandez University, Alicante, Spain; 2 Grupo de Investigación en Terapia Ocupacional (InTeO), Miguel Hernandez University, Alicante, Spain; 3 Alicante Institute for Health and Biomedical Research, Alicante, Spain; 4 Clinical and Epidemiological Neuroscience (NeuroÈpia), Institut d’Investigació Sanitària Pere Virgili (IISPV), Reus, Spain; 5 ISGlobal, Barcelona, Spain; 6 Spanish Consortium for Research on Epidemiology and Public Health (CIBERESP), Madrid, Spain; 7 Environmental Epidemiology and Child Development Group, Biodonostia Health Research Institute, San Sebastian, Spain; 8 School of Psychology, University of the Basque Country, UPV/EHU, San Sebastián, Spain; 9 Centre for Environmental Sciences, Hasselt University, Hasselt, Belgium; 10 Instituto Universitario de Oncología Del Principado de Asturias (IUOPA) – Departamento de Medicina, Universidad de Oviedo, Oviedo, Asturias Spain; 11 Instituto de Investigación Sanitaria Del Principado de Asturias (ISPA), Oviedo, Spain; 12 Servicio de Pediatría, Endocrinología Pediátrica, HUCA, Oviedo, Asturias Spain; 13 Universitat Pompeu Fabra, Barcelona, Spain; 14 Department of Child and Adolescent Psychiatry/Psychology, Erasmus MC, University Medical Centre, Rotterdam, The Netherlands; 15 Sub-Directorate for Public Health and Addictions of Gipuzkoa, Ministry of Health of the Basque Government, Donostia-San Sebastian, Spain

**Keywords:** Telomere length, Neuropsychological function, Working memory, Children

## Abstract

Shortened telomere length (TL) has been associated with lower cognitive performance, different neurological diseases in adults, and certain neurodevelopmental disorders in children. However, the evidence about the association between TL and neuropsychological developmental outcomes in children from the general population is scarce. Therefore, this study aimed to explore the association between TL and neuropsychological function in children 4–5 years of age. We included 686 children from the INMA Project, a population-based birth cohort in Spain. Leucocyte TL was determined by quantitative PCR method, and neuropsychological outcomes were measured using the McCarthy Scales of Children’s Abilities (MCSA). Multiple linear regression models were used to estimate associations adjusted for potential confounding variables. Main findings showed that a longer TL was associated with a higher mean working memory score (β = 4.55; 95% CI = 0.39, 8.71). In addition, longer TL was associated with a higher mean global quantitative score (β = 3.85; 95% CI = −0.19, 7.89), although the association was marginally significant. To our knowledge, this is the first study that shows a positive association between TL and better neuropsychological outcomes in children. Although further research is required to confirm these results, this study supports the hypothesis that TL is essential in protecting and maintaining a child’s health, including cognitive functions such as working memory.

**Supplementary Information:**

The online version contains supplementary material available at 10.1007/s00787-023-02361-y.

## Background

The telomere is a protective non-coding DNA–protein complex at the ends of chromosomes. The primary function of the telomere is to protect and preserve the information in our genome [1, 2]. The telomere length (TL) naturally decreases with age due to cell division processes. However, if the TL becomes critically short, telomeres are recognised as damaged DNA, leading to cell senescence and, consequently, loss of tissue function [3]. Because of this, TL shortening is avowed as one of the key factors of cellular ageing [4]. Although telomeres shorten over the years, there is variability among individuals. Several studies suggest that this could be explained by exposure to different environmental factors that increase oxidative stress and inflammatory processes that accelerate telomere shortening [5–7]. Recent research has observed that the rate of TL attrition is more pronounced during the first years of life [8, 9]. In addition, the intrauterine environment during pregnancy and maternal exposure factors and lifestyles play an important role in the length and maintenance of the telomere of newborns and, therefore, in the potential health outcomes of the child [10–13].

There is scant research exploring the relationship between TL and health problems in childhood, although it has become an area of increasing interest in the last decade [14]. Since TL is considered a promising ageing biomarker, most of the available research has focused on exploring the association between TL and the development of pathologies in adulthood. Some studies showed an association between shorter telomeres and neurological pathologies, such as Alzheimer’s disease [15, 16], and psychiatric pathologies like schizophrenia, bipolar disorder, major depression, anxiety, or post-traumatic stress [17–20]. In the same way, some research observed that longer TL is associated with better performance on general cognitive tests and some cognitive functions, such as attention, processing speed or working memory [21–24]. Some studies have also shown the association between TL and certain neurodevelopmental disorders in the paediatric population, including autism spectrum disorder [25–27] or attention deficit hyperactivity disorder [28–30]. To our knowledge, only one study has explored the association between TL and neuropsychological developmental outcomes at 9 and 30 months, and 5 years of age in children enrolled in the Seychelles Cohort [31]. This study found a slight positive association between cord blood TL and psychomotor outcomes at 30 months, as well as letter-word recognition assessed by the Woodcock-Johnson Achievement Test at the age of five. However, when examining the association with TL at five years, no clear association was observed with various neurodevelopmental functions, including motor skills, language, memory and problem-solving. [31]. It is noteworthy that the limited sample size of 209 children in this previous study might have constrained its ability to detect subtle associations.

Considering the research mentioned above, we hypothesised that children with longer TL would have better neuropsychological functioning. However, it is essential to acknowledge the multifaceted nature of neuropsychological functions, influenced by various factors. Potential effects of TL on these functions might be relatively modest and necessitate a robust statistical power, achievable with larger sample sizes. Consequently, our present study was conceived to address this limitation by examining a substantially larger and more diverse sample of children aged 4–5 years. We acknowledge the intricate nature of the relationship between TL and neuropsychological function, which may exhibit variations across different populations. As such, our research endeavoured to provide a more comprehensive and robust analysis, considering a broader spectrum of influencing factors, to better elucidate these associations. Therefore, the present study aimed to explore the association between TL and neuropsychological functions in children at 4–5 years of age.

## Methods

### Study design and population

This study analysed data from the Infancia y Medio Ambiente (INMA) Project (https://www.proyectoinma.org/). The INMA project is a multicentre and population-based Spanish birth cohort. Its objective is to assess how environmental factors and lifestyles during pregnancy and infancy affect the health and development of children and adolescents. The study population of the INMA project consists of pregnant women from different geographical areas of Spain (Ribera d’Ebre, Menorca, Granada, Valencia, Asturias, Gipuzkoa and Sabadell). Participants were recruited during the first prenatal visit (between 10 and 13 weeks of gestation) at the reference public hospital or the region’s health centre from 2004 to 2008 [32]. The present study included 686 mother–child pairs from Asturias, Gipuzkoa and Sabadell sub-cohorts, who had complete information on the main study variables (TL and neuropsychological development) and potential confounding variables. In addition, parents provided the corresponding informed consent at the beginning of the INMA study. The ethics committee of the Miguel Hernández University of Elche (DPC.ENM.01.20) approved the present study.

## Measures

### Telomere length

Telomere data at 4–5 years was available in Asturias, Gipuzkoa and Sabadell (mean age: 4.4 years, standard deviation (SD) 0.2 years; interquartile range (IR), 4.4–4.5 years). TL was determined using the leukocyte fraction from blood samples collected at the 4-year follow-up visit. DNA was extracted from the buffy coat using the QIAamp DNA Mini Kit (Qiagen) in Asturias, the Flexigen AGKT-WB-640 kit (Qiagen) in Gipuzkoa, and the Chemagen kit (Perkin Elmer) in Sabadell. TL was determined using a modified fluorochrome-based quantitative polymerase chain reaction (qPCR) protocol [33]. Measurements were performed in triplicate on a 7900HT real-time PCR system (Applied Biosystems) in 384-well format. In each cycle, a 6-point serial dilution of a pooled DNA (*n* = 12 DNA samples) was performed to assess qPCR efficiency for telomere (T) and single copy gene (S) runs. The efficiency was 107% for T runs (R^2^ ranged from 0.994 to 0.999) and 97% for S runs (R^2^ ranged from 0.995 to 0.999). TL was analysed using qBase software (Biogazelle, Zwijnarde, Belgium) and expressed as the ratio of telomere copy number to the number of single-copy genes (T/S) relative to the average T/S of the set of all samples. In qBase, TL is calculated as a calibrated normalized relative quantity (CNRQ) [34], which is achieved by first calculating the RQ based on the delta-Cq method for T and S obtained Cq values, using target-specific amplification efficiencies. As the error on the final relative quantities (as a result of the measurement error on the calibrator sample) is very sensitive to the choice of a calibrator sample (sample to which subsequent normalization is applied), normalization was performed to the arithmetic mean quantification values for all analysed samples per cohort, resulting in the NRQ. Moreover, as samples per cohort were measured over multiple qPCR plates, 8 inter-run calibrators (IRC’s) were used to calculate an additional correction factor to eliminate run-to-run differences, obtaining the final T/S ratio (CNRQ). Mathematical formulas to calculate RQ, NRQ and CNRQs are provided by Hellemans et al. [34]. To check the reliability/accuracy of the applied protocol, the intraclass correlation coefficients (ICC) of triplicate measures for T values (0.957; 95%CI: 0.954 to 0.96; *P* < 0.0001), S values (0.968; 95%CI: 0.965 to 0.97; *P* < 0.0001) and T/S ratio’s (0.925; 95%CI: 0.918 to 0.93; *P* < 0.0001) were estimated, using the ICC R-code provided by the Telomere Research Network [35]. In addition, based on the 8 IRC’s ran over all the qPCR plates, an inter-assay ICC was calculated (0.898; 95%CI: 0.77 to 0.948; *P* < 0.0001). Based on the standard curves qPCR efficiency for T runs was 107 on average. Further details on the TL determination process are available in the Supplementary material.

### Neuropsychological functions

Neuropsychological functions were assessed using the standardised version adapted to the Spanish population of the McCarthy Scales of Children’s Abilities (MSCA), which evaluates children’s cognitive and motor development [36]. Children from the Gipuzkoa region were assessed using the Basque version of the instrument (MSCA-E) [37]. Trained psychologists administered the neuropsychological scales to the children at the follow-up visit at 4–5 years of age. The MSCA consists of 6 scales: general cognitive, verbal, perceptive-performance, quantitative, memory, and motor functions. In the present study, we also used 11 scales created by Julvez and colleagues [38], which were derived from the original MSCA items. These new scales evaluate the following neuropsychological functions: executive functions, including visual and verbal processes; visual and verbal span; working memory; verbal memory; gross and fine motor functions; and cognitive functions of the posterior cerebral cortex, including verbal (left cortex) and visual (right cortex) functions. To ensure the reliability of the tests, we estimated the interrater reliability by applying intraclass correlation. MSCA global scales, including general cognitive, verbal, perceptive-performance, quantitative, memory, and motor functions, obtained coefficients of 0.97, 0.98, 0.78, 0.98, 0.96, and 0.92, respectively. In addition, the internal consistency of the global scales, measured by the Cronbach’s alpha coefficient, was 0.90, 0.79, 0.81, 0.77, 0.74, and 0.64 for general cognitive, verbal, perceptive-performance, quantitative, memory, and motor functions, respectively. New MSCA subscales displayed a moderately good alpha coefficient (i.e., ⪰0.70) overall [39]. To homogenise test scores, we standardised the MSCA raw scores to a mean of 100 and a standard deviation of 15. Higher scores on the different scales of the test indicate better neuropsychological functioning in children.

### Potential confounders

We selected potential confounders based on existing literature, encompassing sociodemographic characteristics, environmental exposures, and lifestyles variables that could plausibly b influence both TL and the neuropsychological functions in children [40–43]. Main regression models were adjusted for the following covariates: variables measured during pregnancy, such as maternal educational level (categorised as primary, secondary or university), parity (0 or ≥ 1 deliveries), and environmental NO_2_ concentrations (expressed in μg/m^3^); and variables collected at the 4–5-year follow-up visit, including child’s age at the time of the neuropsychological assessment (in years), the relative Mediterranean diet score (continuous) [44], daily energy intake (in kilocalories), and the date of the blood sample extraction for telomere determination categorised into the seasons of the year (spring, summer, autumn, or winter). In addition, we accounted for the geographical study area (i.e., Asturias, Gipuzkoa or Sabadell).

## Data analysis

All statistical analyses were performed with R software version 4.1.2 (R Foundation for Statistical Computing). A statistical significance level was established at 0.05, and all contrasts were bilateral. Normal distribution of continuous variables was checked by using the Kolmogorov–Smirnov test with Lilliefors correction.

A descriptive analysis of the maternal and child characteristics by cohort was performed using frequencies and percentages (%) for categorical variables and median and interquartile range (IR) for continuous variables since they were not normally distributed. We explored differences between participant characteristics and cohorts using the chi-square test (categorical variables) and Kruskal–Wallis test (continuous variables).

Multiple linear regression models were used to estimate associations between TL and neuropsychological function outcomes. To control potential confounding, separate models were built for each neuropsychological function, including covariates in the descriptive analysis with *p* < 0.2 and/or if they changed the magnitude of the main effect by ≥ 10%. Heterogeneity between cohorts was quantified using the I^2^ statistic. We applied meta-analytic techniques to obtain pooled estimates under the fixed-effects hypothesis (I^2^ < 50%) or random-effects models when detecting heterogeneity (I^2^ > 50%) [45]. No multiple testing corrections were made due to the exploratory nature of the present study.

Finally, a sensitivity analysis was conducted to assess the significant findings’ robustness making a set of assumptions by using variables that could likely be related to either TL or child neuropsychological function [46–52]. First, we examined the effect of the mother’s verbal reasoning (measured by the similarities test of the Wechsler Adult Intelligence Scale III) and different child’s lifestyles, such as oily fish consumption (grams), television watching (hours per week), and sleep duration (hours per day) separately as a further adjustment to the main model. Second, we ran the main model excluding children with low scores on the MCSA (*n* = 35). And third, we checked if there were changes in the main association stratifying models by sex (boys or girls) and by comparing values below or above the median of the level of inflammation as determined by C-reactive protein (quantiles), abdominal circumference (centimetres), and body mass index (kg/m^2^) of the children at 4–5 years of age.

## Results

Table 1 shows the sociodemographic characteristics, environmental exposure factors, and lifestyles of the mothers and their children. Of the 686 participating women, 41.5% were from the Asturias region, 39.1% and 19.4% resided in Sabadell and Gipuzkoa, respectively. In general, we observed that mothers in the Gipuzkoa region had a higher educational level than women in the other study cohorts. In addition, they had on average lower exposure to the ambient pollutant NO_2_ during their pregnancy (median = 15.1 μg/m^3^, IR = 13.9–16.9) compared to mothers from Asturias (median = 27.6, IR = 16.8–37.6) and Sabadell (median = 39.0, IR = 30.3–44.0), who had higher exposure.

**Table 1:** Socio-demographic characteristics and lifestyles in mother**s** and children at 4–5 years of the INMA project, Spain (*n* = 686)

	All cohorts (n = 686)	Asturias (n = 285)	Gipuzkoa (n = 133)	Sabadell (n = 268)	P^a^
*Characteristics of mother’s*					
Educational level, n (%)					
Primary or less	134 (19.5)	46 (16.1)	21 (15.8)	67 (25.0)	0.001
Secondary	285 (41.6)	128 (44.9)	44 (33.1)	113 (42.2)	
University	267 (38.9)	111 (39.0)	68 (51.1)	88 (32.8)	
Parity ≥ 1, n (%)	293 (42.7)	110 (38.6)	64 (48.1)	119 (44.4)	0.144
NO_2_ μg/m^3^ during pregnancy, median (IR)	28.7 (16.7–40.5)	27.6 (16.8–37.6)	15.1 (13.9–16.9)	39.0 (30.3–44.0)	< 0.001
*Characteristics of children*					
Age in years at MCSA examination, median (IR)	4.4 (4.3–4.5)	4.4 (4.3–4.5)	4.4 (4.4–4.5)	4.4 (4.4–4.5)	0.102
Daily energy intake in kcals/day, median (IR)	1579.6 (1390.0–1822.6)	1618.5 (1428.5–1868.3)	1464.5 (1277.6–1695.5)	1598.5 (1430.7–1819.3)	< 0.001
Relative Mediterranean diet score, median (IR)	9.0 (7.0–10.0)	9.0 (8.0–11.0)	8.0 (6.0–10.0)	8.0 (6.0–10.0)	< 0.001
Telomere length, median (IR)	1.0 (0.9–1.2)	1.0 (0.9–1.1)	1.2 (1.0–1.4)	1.0 (0.9–1.1)	< 0.001
Season of blood extraction, n (%)					
Spring	185 (27.0)	84 (29.5)	16 (12.0)	85 (31.7)	< 0.001
Summer	174 (25.4)	31 (10.9)	83 (62.4)	60 (22.4)	
Autumn	154 (22.4)	74 (25.9)	18 (13.6)	62 (23.1)	
Winter	173 (25.2)	96 (33.7)	16 (12.0)	61 (22.8)	

*IR* interquartile range, *MCSA* McCarthy scales of children’s abilities, *INMA* Infancia y Medio Ambiente

^a^The chi-square test was used for categorical variables and the Kruskall-Wallis test for continuous nonparametric variables

Regarding children, those from Gipuzkoa had a lower energy intake per day (median = 1464.5 kcals/day, IR = 1277.6–1695.5) and a longer TL on average (median = 1.2, IR = 1.0–1.4) than those from Asturias and Sabadell. In addition, the relative Mediterranean diet score was higher in children from Asturias (median = 9, IR = 8–11) than those from Gipuzkoa and Sabadell (median = 8, IR = 6–10). Differences were also observed in the date of blood sample extraction for telomere determination. In Gipuzkoa, the blood extraction was primarily performed in summer (62.4%); in Asturias, it was carried out roughly in the same proportion in spring, autumn, and winter, and, to a lesser extent, in summer. In Sabadell, the blood samples were roughly equal in numbers across the four seasons.

Table 2 presents the results of multiple linear regression models exploring the association between TL and children’s neuropsychological functions at 4–5 years of age, adjusted for potential confounding variables. In the MCSA areas, we observed that longer TL was associated with a higher mean global quantitative score, although the association was marginally significant (β = 3.85; 95% CI = −0.19, 7.89; *p* = 0.062; I^2^ = 0.0%). Regarding MCSA functions, longer TL was positively and statistically significantly associated with a higher mean working memory score (β = 4.55; 95% CI = 0.39, 8.71; *p* = 0.032; I^2^ = 0.0%). However, no associations were obtained between TL and the rest of the neuropsychological functions of children at this age.

**Table 2:** Association between leukocyte telomere length and neuropsychological function of children at 4–5 years of age in the INMA project, Spain (*n* = 686)

	LTL (n = 686)		
Neuropsychological function	β (95% CI)	*p*	I^2^ (%)^b^
*MCSA areas*^a^			
General cognitive index	−0.54 (−4.37, 3.30)	0.783	19.3
Global verbal	−2.79 (−6.70, 1.41)	0.193	0.0
Global perceptual-performance	1.98 (−1.92, 5.89)	0.319	49.8
Global quantitative index	3.85 (−0.19, 7.89)	0.062	0.0
Global memory	−0.44 (−4.63, 3.73)	0.833	38.1
Global motor skills	0.43 (−3.84, 4.71)	0.843	0.0
*MCSA functions*^a^			
Executive	0.56 (−3.36, 4.48)	0.779	0.0
Visual executive	0.05 (−4.10, 4.21)	0.979	27.2
Verbal executive	0.75 (−3.21, 4.17)	0.710	0.0
Visual and verbal span	−0.81 (−7.19, 5.56)	0.803	53.1
Working memory	**4.55 (0.39, 8.71)**	**0.032**	0.0
Verbal memory	−2.40 (−6.89, 2.08)	0.294	0.0
Gross motor	−2.35 (−6.67, 1.96)	0.285	0.0
Fine motor	3.03 (−1.28, 7.34)	0.168	0.0
CFPC	−0.01 (−4.00, 3.99)	0.997	40.0
CFLPC (verbal)	−2.59 (−6.92, 1.73)	0.240	0.0
CFRPC (visual)	2.91 (−1.27, 7.10)	0.173	27.7

*LTL* leukocyte telomere length, *MCSA* McCarthy scales of children’s abilities, *CFCP* cognitive function of posterior cortex, *CFLPC* cognitive function of left posterior cortex, *CFRPC* cognitive function of right posterior cortex, *CI* confidence interval, *INMA* Infancia y Medio Ambiente

^a^All linear regression models were adjusted by mother’s educational level (primary or less, secondary, or university), parity (0 or ≥ 1), NO_2_ exposure in pregnancy (μg/m^3^), child’s age at MCSA examination (in years), child’s relative Mediterranean diet score (continuous), energy intake (in kilocalories/day) and season of blood extraction (spring, summer, autumn or winter) at 4–5 years

^b^We used the results from the fixed-effects meta-analysis model when < 50% and from the random-effects meta-analysis model when > 50%

**Fig. 1:**
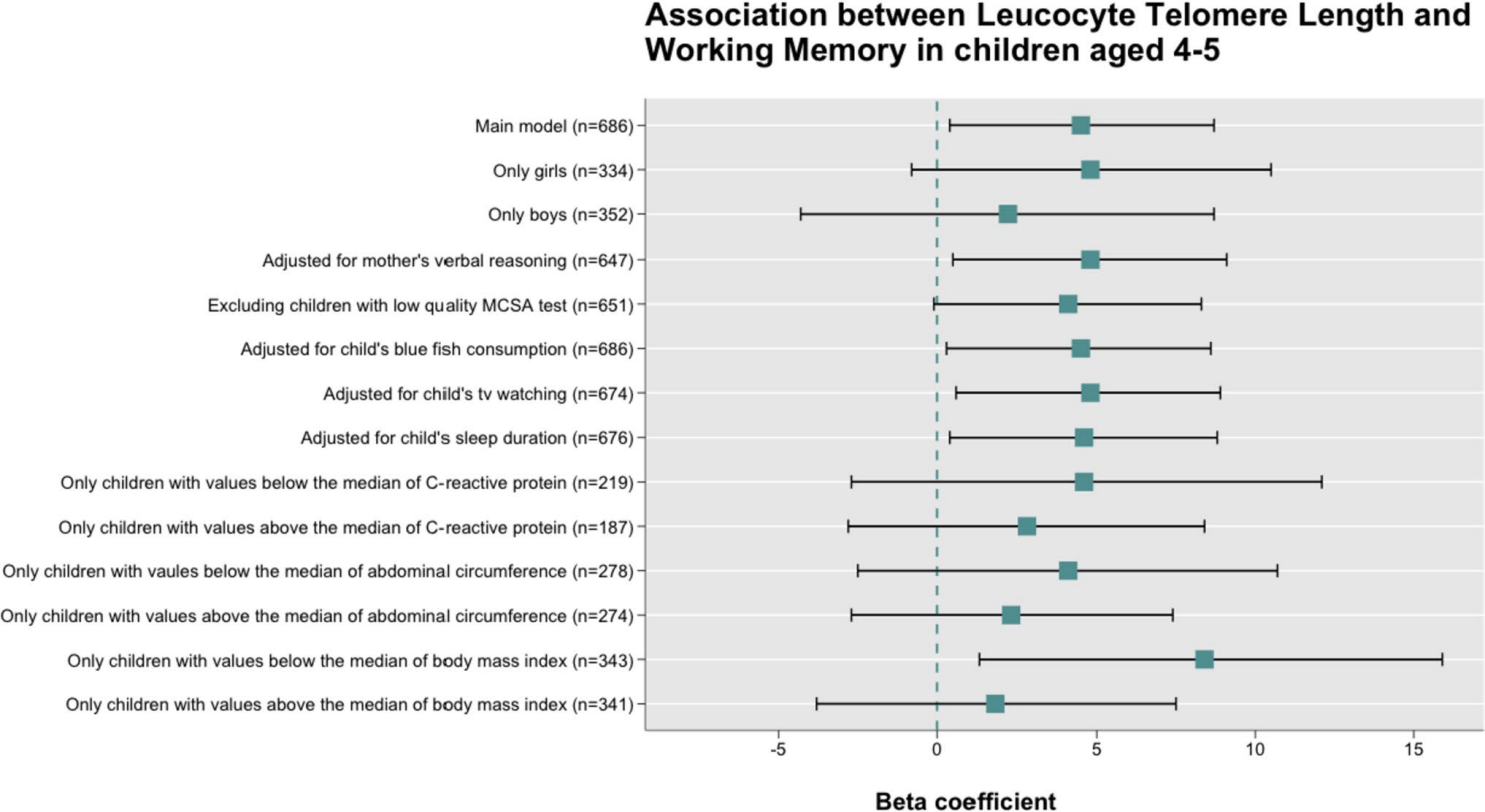
Sensitivity analysis of the association between Leucocyte Telomere Length and Working Memory in children aged 4–5

The results of the sensitivity analysis of the association between TL and working memory function are presented in Fig. 1. After adjusting the main model by mother’s verbal reasoning (β = 4.83; 95% CI = 0.50, 9.15; *p* = 0.028) and children’s lifestyles, including fish consumption (β = 4.46; 95% CI = 0.28, 8.64; *p* = 0.036), television watching (β = 4.76; 95% CI = 0.58, 8.94; *p* = 0.026) and sleep duration (β = 4.64; 95% CI = 0.43, 8.85; *p* = 0.031), the positive significant association observed for working memory (β = 4.55; 95% CI = 0.39, 8.71; *P* = 0.032) remained almost the same. We also observed no changes when excluding children who scored low on the MCSA, although the association became marginally significant (β = 4.12; 95% CI = −0.10, 8.34; *p* = 0.055). However, when we redid the analysis stratifying the main model by sex, we observed the association for boys dropped by 2 points (β = 2.16; 95% CI = −4.34, 8.67; *p* = 0.515) compared to girls (β = 4.81; 95% CI = −0.85, 10.47; *p* = 0.096), although the association was not statistically significant. The association for children with values above the median of C-reactive protein (β = 2.78; 95% CI = −2.84, 8.41; *p* = 0.334), abdominal circumference (β = 2.34; 95% CI = −2.73, 7.41; *p* = 0.368), and body mass index (β = 1.85; 95% CI = −3.76, 7.46; *p* = 0.518) decreased considerably although the statistical significance was not reached. In contrast, we did not observe substantial changes for the effect of children with values below the median of C-reactive protein (β = 4.68; CI 95% = −2.75, 12.11; *p* = 0.217) and abdominal circumference (β = 4.08; 95% CI = −2.50, 10.67; *p* = 0.224). However, the estimate for children with body mass index below the median increased the effect considerably (β = 8.37; 95% CI = 1.32, 15.91; *p* = 0.020) and remained statistically significant.

## Discussion

The results obtained in the present study suggest a potential association between longer TL in childhood and enhanced working memory performance. Additionally, our findings hint at a possible influence of TL on the mean score of the quantitative index assessed by the MCSA scales. To our knowledge, this is the first time TL is associated with better neurocognitive outcomes in children aged 4–5 years. However, while our findings provide initial insights, it is essential to approach these results with caution and consider them as a starting point for further research into the intricate interplay between TL and cognitive functions during early childhood.

Our results are not directly comparable with the findings obtained from a previous study conducted on 5-year-old children without pathology of a birth cohort from Seychelles [31]. Both studies share a similar epidemiological study design, but they differ in the tests used to assess neuropsychological outcomes. In contrast to our study, Feiler and colleagues (2018) [31] did not include the assessment of the working memory function and quantitative abilities, which showed a positive effect of longer TL in our research. However, they examined the association between TL and neurodevelopmental functions, such as motor function, language, and math problem-solving, broadly similar to those we measured. In this respect, both studies obtained no conclusive associations for these functions. Another aspect that should be considered is that the average TL of the children differed among studies, although TL was determined using the same method (qPCR) at almost the same age. Children in our study presented a longer average TL than children from the Seychelles Child Development Study [31] (1.0 vs 0.71, respectively). Nevertheless, the underlying explanation by which longer TL may be potentially associated with better working memory or numerical tasks at the age of 4–5 remains unclear, requiring further investigation.

To the best of our knowledge, no previous studies have specifically examined the association between TL and working memory in the paediatric population, and only limited data are available from studies conducted in adults. For example, a study of 382 healthy women [21] showed a positive correlation between TL and performance on a functional memory test, although statistical significance was not reached. In a more recent study involving 325 African-American and white adults, an association was observed between shorter TL and poorer performance on a working memory test. However, this association was only statistically significant in white participants [24].

In the absence of definitive evidence, our finding that longer TL may be associated with enhanced working memory in children could be attributed to the role of TL in maintaining cellular stability and, by extension, cellular stability of the brain by protecting neuronal cells from cell apoptosis [1, 2, 53]. Recent insights from a meta-analysis of 27 studies on the impact of TL in brain ageing [54] suggested that telomeres may have a positive influence on brain morphology and cognition, potentially leading to larger brain size and improved connectivity. Although statistical significance was not reached for both girls and boys because of limited statistical power, our findings of sex differences are in line with this study that suggests that longer telomeres may have a more pronounced beneficial effect on women compared to men [54]. Recent insights into the mechanistic basis of the potential sex differences in neurobiology have suggested that hormones, particularly oestradiol, may be partly responsible for changes in brain functions, brain health, and the molecular functioning of brain cells [46]. While these insights are primarily derived from experimental animal studies, they postulate that oestradiol may serve a crucial neuroprotective function in the brain, especially in memory, suggesting that this oestrogen may trigger different cell-signalling pathways in males and females. In parallel, another likely explanation for the positive association between TL and working memory could be attributed to the fact that basic cognitive functions such as working memory or attention appear to be more vulnerable to the ageing process and/or molecular and cellular damage than complex cognitive functions such as long-term memory, executive function, or global cognition [53, 55, 56]. As such, it is plausible to assume that the protective effect of a longer TL could presumably be more significant for these basic cognitive functions rather than complex ones. Interestingly, given that working memory deficits have been associated with a decline in brain structural integrity, working memory has been proposed as a valuable marker for identifying potential age-related cognitive decline [57]. In addition, the association between TL and the global numerical area, while not reaching conventional levels of statistical significance, provides valuable support for the observed association with working memory. This connection is particularly pertinent because numerical skills assessed by the MCSA quantitative scale place a notable demand on working memory resources. Notably, previous research indicated that children encountering challenges in mathematical operations often showed deficits in the working memory domain [57].

The present study’s findings also showed that the level of inflammation might influence the effect of TL on working memory at 4–5 years of age. This result is consistent with previous research showing that an elevated level of pro-inflammatory cytokines, like C-reactive protein, was associated with poorer working memory in children and young individuals [48, 49]. At the neurobiological level, this association is supported by the fact that inflammation processes were found to cause changes in neural circuitry, neurotransmitter activity, the neuroendocrine system and neurogenesis, impacting brain function and, by extension, cognitive functions [47, 48, 58, 59]. Moreover, it should be noted that TL attrition has also been associated with inflammation events, including higher body mass index or biomarkers such as C-reactive protein [50], suggesting that inflammatory events might involve an interplay between telomeric dysfunction and poor cognitive functioning.

While our study offers valuable insights into the association between TL and cognitive function in young children, it is important to acknowledge both limitations and strengths of our research. The INMA project is a population-based follow-up study that collected extensive information (e.g., socio-demographics, lifestyles, environmental pollutants, etc.) during pregnancy and postnatal follow-up visits. This extensive dataset minimises the possible occurrence of recall and sample selection biases, thus enhancing the robustness of our findings. The prospective nature of this study allowed us to account for a wide range of confounding factors and modifiers, although residual confounding, unknown factors or modifiers cannot be dismissed. Regarding neuropsychological assessment, we used the standardised Spanish and Basque versions of the MCSA applied by psychologists trained in administering the test, ensuring test validity and quality of the information. However, we should not disregard the multiple testing problem since numerous tests were applied in this study, the potential for Type I errors emerges as a significant concern and should be contemplated in interpreting the results. Moreover, TL was determined using quantitative PCR methods following a strict protocol. Researchers commonly use PCR because it is inexpensive and fast, although this methodology provides an average measurement of all samples, probably introducing measurement errors that warrant caution when analysing the data [60]. As an observational cross-sectional study, our findings offer valuable insights into the potential associations between TL and cognitive function in young children. However, the cross-sectional design inherently limits our capacity to establish causal relationships. Fortunately, the prospective design of the INMA project will allow us to establish a temporal sequence of events through follow-up assessments and confirm long-term effects. It is important to note that this study primarily focused on the influence of environmental factors, including prenatal and postnatal exposures, on TL in young children aged 4–5. While genetic factors play a significant role in TL heritability and regulation, they were not the primary focus of our investigation. Our study primarily aimed to explore the potential impact of specific environmental factors on TL. Future research efforts with a more comprehensive examination of genetic determinants and their interaction with environmental exposures will be essential to provide a more holistic understanding of TL dynamics in children. Despite the shortcomings, this study may contribute to the relatively limited body of knowledge in this specific area of research, enriching the scientific understanding of TL’s role in cognitive function in early childhood. These findings not only provide a foundation for future investigations but also underscore the necessity for further research involving larger and more diverse cohorts to confirm and extend our results.

In conclusion, the results of the present study offer valuable insights into the possible association between TL and working memory in 4–5-year-olds. While the results indicate a positive trend between TL and the quantitative index, this association did not reach conventional levels of statistical significance. No association was observed between TL and the rest of the MSCA scales. Further research is essential to confirm these findings and uncover the biological pathways by which the telomere length is associated with cognitive functions such as working memory during childhood. Nonetheless, we believe this study contributes to the body of evidence supporting that TL plays an important role in preserving health and, by extension, it may have a positive impact on children’s neuropsychological development.

### Supplementary Information

Below is the link to the electronic supplementary material.

Supplementary file1 (DOCX 21 KB)
