# Supplementary material for: Association between telomere length and neuropsychological function at 4–5 years in children from the INMA project: a cross-sectional study
Source: Eur Child Adolesc Psychiatry. 2024 Jan 22;33(8):2803–12. doi: 10.1007/s00787-023-02361-y (PMC11272730; doi:10.1007/s00787-023-02361-y)
Supplement: Supplementary file 1 — Supplementary file1 (DOCX 21 KB) [file 787_2023_2361_MOESM1_ESM.docx]

**Supplementary material**

**Average relative telomere length measurement using qPCR.**

Average relative telomere length was measured using a modified qPCR protocol in accordance with Cawthon et al. [1] that was reported by Martens and colleagues [2]. Firstly, DNA quantity and purity was assessed using a Nanodrop 1000 spectrophotometer (Isogen, Life Science, Belgium) considering the DNA pure when the A260/280 was greater than 1.80 and A260/230 greater than 2.0. DNA integrity was assessed by agarose gel-electrophoresis. To ensure a uniform DNA input of 5 ng for each qPCR reaction, samples were diluted and checked using the Quant-iT™ PicoGreen® dsDNA Assay Kit (Life Technologies, Europe). The samples All samples were measured in triplicate using a 7900HT Fast RealTime PCR System (Applied Biosystems) in a 384-well format. The reaction mixture used to assess telomeres contained 1x QuantiTect SYBR Green PCR master mix (Qiagen, Inc., Venlo, the Netherlands), 2 mM dithiothreitol (DTT), 300 nM telg primer (ACACTAAGGTTTGGGTTTGGGTTTGGGTTTGGGT TAGTGT) and 900 nM telc primer (TGTTAGGTATCCCTATCCCTATCCCTATCCCTATCCCTAACA). The applied cycling conditions were: 1 cycle at 95°C for 10 min, 2 cycles at 94°C for 15 sec and 49°C for 2 min, and 30 cycles at 94°C for 15 sec, 62°C for 20 sec, and 74°C for 1 min and 40 sec. The single-copy gene (human β globin) qPCR mixture contained 1x QuantiTect SYBR Green PCR master mix, 400 nM HBG1 primer (GCTTCTGACACAACTGTGTTCACTAGC) and 400 nM HBG2 primer (CACCAACTTCATCCACGTTCACC). The same cycling conditions were used: 1 cycle at 95°C for 10 min, 40 cycles at 95°C for 15 sec, and 58°C for 1 min and 20 sec. After PCR cycling individual qPCR curves and melt curves were visually inspected and when a run error was observed the Cq value was removed from subsequent analysis. In addition, when triplicate measures showed a deviation of more than 0.3 in Cq value these were removed from subsequent analysis.

**References.**

1. Cawthon RM. Telomere length measurement by a novel monochrome multiplex quantitative PCR method. *Nucleic Acids Res*. 2009;37(3):e21. doi:10.1093/nar/gkn1027

2. Martens DS, Van Der Stukken C, Derom C, Thiery E, Bijnens EM, Nawrot TS. Newborn telomere length predicts later life telomere length: Tracking telomere length from birth to child- and adulthood. *EBioMedicine*. 2021;63:103164. doi:10.1016/j.ebiom.2020.103164
